# WIPI-Mediated Autophagy and Longevity

**DOI:** 10.3390/cells4020202

**Published:** 2015-05-22

**Authors:** Mona Grimmel, Charlotte Backhaus, Tassula Proikas-Cezanne

**Affiliations:** Autophagy Laboratory, Department of Molecular Biology, Interfaculty Institute of Cell Biology, Faculty of Science, Eberhard Karls University Tuebingen, Auf der Morgenstelle 15, 72076 Tuebingen, Germany

**Keywords:** autophagy, longevity, WIPI, WIPI-1, WIPI-2, ATG-18, EPG-6, *C. elegans*

## Abstract

Autophagy is a lysosomal degradation process for cytoplasmic components, including organelles, membranes, and proteins, and critically secures eukaryotic cellular homeostasis and survival. Moreover, autophagy-related (ATG) genes are considered essential for longevity control in model organisms. Central to the regulatory relationship between autophagy and longevity is the control of insulin/insulin-like growth factor receptor-driven activation of mTOR (mechanistic target of rapamycin), which inhibits WIPI (WD repeat protein interacting with phosphoinositides)-mediated autophagosome formation. Release of the inhibitory mTOR action on autophagy permits the production of PI3P (phosphatidylinositol-3 phosphate), predominantly at the endoplasmic reticulum, to function as an initiation signal for the formation of autophagosomes. WIPI proteins detect this pool of newly produced PI3P and function as essential PI3P effector proteins that recruit downstream autophagy-related (ATG) proteins. The important role of WIPI proteins in autophagy is highlighted by functional knockout of the WIPI homologues ATG-18 and EPG-6 in *Caenorhabditis elegans* (*C. elegans*). Adult lifespan is significantly reduced in ATG-18 mutant animals, demonstrating that longevity as such is crucially determined by essential autophagy factors. In this review we summarize the role of WIPI proteins and their *C. elegans* homologues with regard to the molecular basis of aging. As the development of strategies on how to increase health span in humans is increasingly appreciated, we speculate that targeting WIPI protein function might represent a therapeutic opportunity to fight and delay the onset of age-related human diseases.

## 1. Introduction

The process of aging has always been a topic of fundamental interest in life science and biomedical research. Aging is considered to be the loss of physiological integrity accompanied by a cumulative dysfunction in securing cellular homeostasis, resulting in the accumulation of damage and a progressive decline of cellular function over time. As life expectancy rises, it is becoming increasingly important to extend the time span of healthy aging, the so-called health span, in human. Whether or not it will become possible to delay or restore age-related cellular alterations to counteract age-related human diseases depends on the further understanding of the genetic basis of longevity.

Studying long- and short-lived mutant model organisms, including nematodes, flies, and mice, provided the first insights into molecular pathways that determine longevity [1,2,3]. However, how context-dependent molecular networks contribute to the progression of aging remains unclear. Nevertheless, the following genetic pathways and biochemical processes that secure the homeostasis of eukaryotic cells and organisms are considered hallmarks of aging: (a) genomic instability, (b) telomere attrition, epigenetic alterations, (c) loss of proteostasis, (d) deregulated nutrient-sensing, (e) mitochondrial dysfunction, (f) cellular senescence, (g) stem cell exhaustion, and (h) altered intercellular communication [4]. Importantly, an age-dependent decline in autophagic activity contributes to the age-associated perturbation of proteostasis. Moreover, the functional decline of autophagy represents not only a characteristic feature of aging, but also the basis for the onset of age-related human diseases and aging as such [4].

Autophagy is an intracellular bulk degradation process evolutionarily conserved across all eukaryotes. The process of autophagy permits both the stochastic turnover of cytoplasmic material, including proteins, lipids, and organelles, as well as the specific degradation of aberrant cellular structures, including protein aggregates, damaged mitochondria, and invaded pathogens. Components degraded through autophagy provide building blocks for cellular storage and anabolic recycling mechanisms. Basal autophagy occurs constitutively on a low level, but upon a great variety of cellular insults, such as starvation, the process of autophagy is induced and engaged to compensate for nutrient and energy shortages. As a result, autophagy clears the cytoplasm of superfluous and harmful material and fights the onset of age-related human diseases, including neurodegeneration and cancer [5,6]. Although autophagy dysfunction is considered to contribute to the initiation and development of age-related human diseases and aging, the precise role of autophagy-related (ATG) genes in the process of aging is unclear and thought to function in a strongly context-dependent manner [7,8]. In order to further understand the contribution of autophagy in the determination of aging and longevity, both autophagic and non-autophagic functions of the ATG machinery need to be recognized. Central to this understanding is the molecular contribution of each individual ATG gene to the formation of autophagosomes, a distinct feature of the process of autophagy.

**Figure 1:**
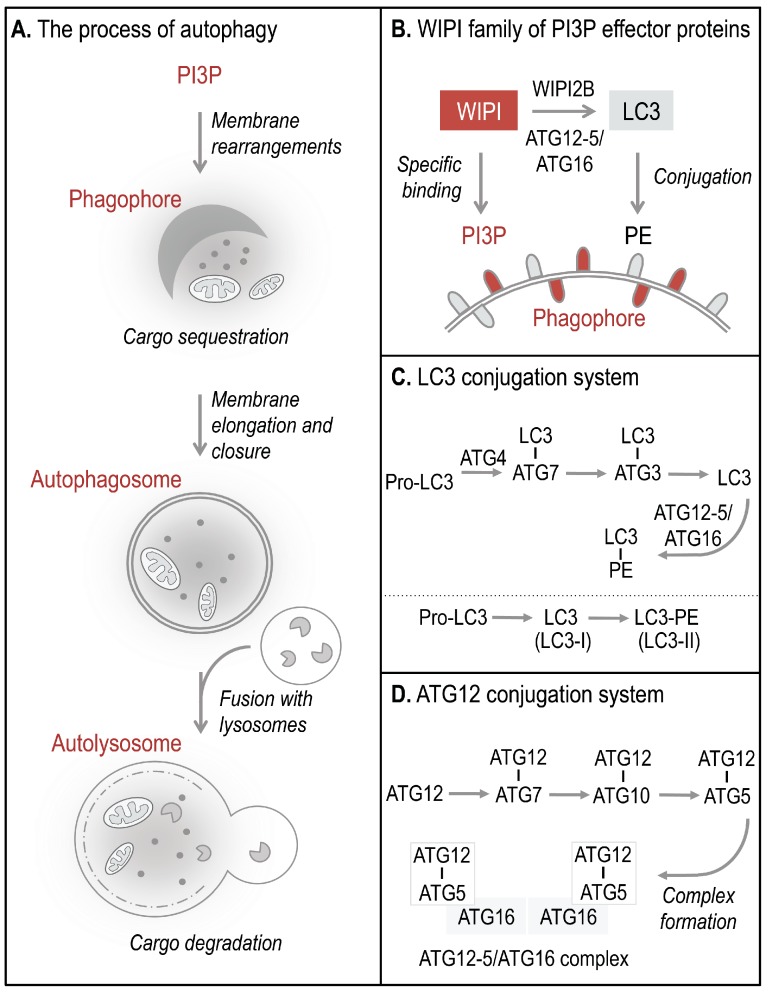
The process of WIPI-mediated autophagy. The process of autophagy is initiated by ER-localized production of PI3P leading to the formation of a template membrane, referred to as a phagophore (or isolation membrane) by unknown steps of membrane rearrangements (**A**). The phagophore elongates by sequestering cytoplasmic cargo, including proteins, lipids, membranes, and organelles, and closes to form a double-membrane vesicle called an autophagosome. Subsequently, the autophagosome delivers the sequestered cargo to the lysosomal compartment for final degradation. During this process the autophagosome fuses with lysosomes to form so-called autolysosomes, where the inner autophagosomal membrane and the cargo are rapidly degraded and the components are released into the cytosol for subsequent recycling or storage purposes. WIPI proteins function as essential PI3P-effector proteins at the nascent autophagosome (**B**). WIPI proteins specifically bind to PI3P and WIPI2B promotes the conjugation of LC3 to PE (LC3 lipidation) through the recruitment of the ATG12-5/ATG16 complex. LC3 lipidation is initiated by C-terminal cleavage of pro-LC3 by ATG4, which becomes conjugated to PE via ATG7 and ATG3 (**C**). Conjugation of LC3 to PE, (LC3-PE, also referred to as LC3-II) requires the ATG12-5/ATG16 complex. ATG12-5/ATG16 complex formation is initiated by the conjugation of ATG12 to ATG5 (ATG12-5), via ATG7 and ATG10 (**D**). Subsequently, ATG16 associates with ATG12-5 to form the multiprotein ATG12-5/ATG16 complex.

**Figure 2:**
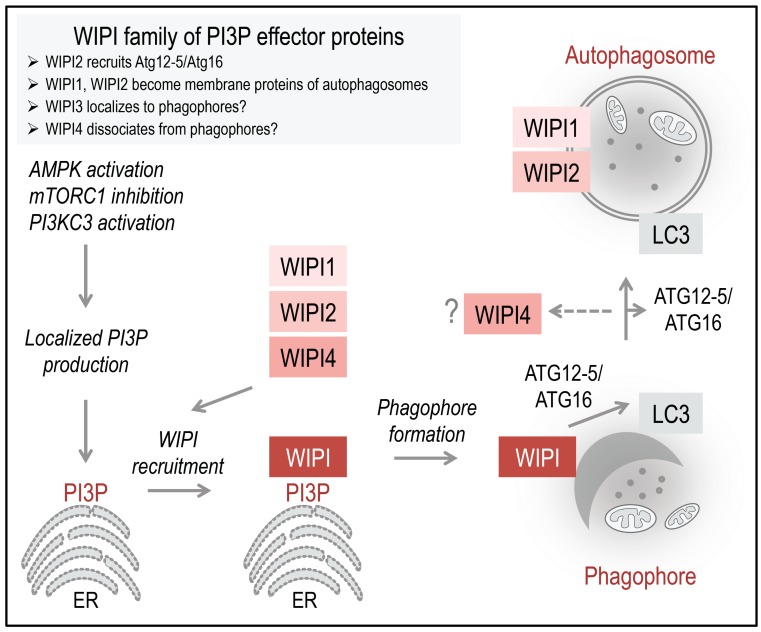
Working model: The PI3P-effector function of human WIPI proteins at the nascent autophagosome. Initiation of ER-localized PI3P production is regulated by the differential actions of AMPK and mTORC1 on the ULK1 complex. Following AMPK-mediated ULK1 activation, the lipid kinase PI3KC3, in complex with Beclin 1, ATG14, and Vps15, is activated to produce PI3P. Upon PI3P production, WIPI proteins are rapidly recruited to the site of autophagosome formation, where WIPI proteins specifically bind PI3P and permit the subsequent formation and proper elongation of the phagophore, as suggested for WIPI1, WIPI2, and WIPI4. Here, WIPI2B recruits the ATG12-5/ATG16 complex via binding to ATG16, permitting LC3 conjugation to PE. It is unknown whether or not WIPI3 is recruited to the phagophore. WIPI1 and WIPI2 have been detected at both the inner and outer membrane of autophagosomes. WIPI1 was further shown to colocalize with the lysosomal protein LAMP1; however, WIPI2 was not found to colocalize with LAMP2. WIPI4 might dissociate in the process of autophagosome closure and maturation. The schematic drawings of the ER was obtained from Motifolio.

Autophagosomes, double-membrane vesicles that are formed to sequester and deliver cytoplasmic material for lysosomal degradation, derive from the elongation and closure of a membrane precursor, referred to as an isolation membrane or phagophore (Figure 1A) [5]. The process of autophagy is executed by a set of essential ATG proteins, originally identified in yeast, collectively functioning in precise hierarchies [9] during canonical and non-canonical pathways that control and execute autophagy [10]. During the process of phagophore elongation and autophagosome closure, phosphoinositide 3-kinase class III (PI3KC3) phosphorylates phosphatidylinositol (PI) to generate phosphatidylinositol 3-phosphate (PI3P) [5]. PI3P production represents the initiation signal for autophagosome formation regulated by both canonical and non-canonical autophagy pathways. The four members of the human WIPI protein family (WIPI1-4) are considered to decode the PI3P signal upon specific binding of WIPI proteins to PI3P at the nascent autophagosome (Figure 1B, 2) [11,12]. One of the essential PI3P-effector functions of WIPI2B is the recruitment of the ATG12-5/ATG16 complex that conjugates LC3 to phosphatidylethanolamine (PE) (Figure 1B–D) for subsequent phagophore elongation [13]. Conjugation of LC3 to PE at the phagophore results from the action of two autophagy-specific ubiquitin-like conjugation systems, the LC3 (Figure 1C) and ATG12 (Figure 1D) conjugation system [9]. In order to conjugate LC3 to PE, the C-terminal end of pro-LC3 is cleaved off and exposes a conserved glycine residue used for conjugation to ATG7, followed by the conjugation to ATG3 (Figure 1C). Finally, LC3 is conjugated to PE (LC3 lipidation) and is thereafter referred to as LC3-II (or LC3-PE), the membrane-bound form of LC3 (Figure 1B). LC3 lipidation is executed by a product of the ATG12 conjugation system, the ATG12-5/ATG16 complex (Figure 1D) [9,10,14]. Functionally, membrane-bound LC3 is involved in cargo recognition and hemifusion with incoming membranes to permit phagophore elongation [14,15].

*C. elegans*, an invaluable animal model commonly employed to decipher the molecular and genetic basis for longevity and aging, exhibits two WIPI homologues, ATG-18 with sequence similarity to WIPI1 and WIPI2, and EPG-6, with sequence homology to WIPI3 and WIPI4 [11,16,17]. An overview of studies conducted on the function of ATG-18 and EPG-6 in *C. elegans* is given in Table 1. It was early recognized that functional inactivation of ATG-18 through genomic atg-18 mutation significantly decreases the lifespan of *C. elegans* and causes premature appearance of age-related locomotory defects (Table 1) [18]. Additionally, suppression of ATG-18 function through *atg-18* RNAi treatments also reduced the lifespan of *C. elegans* [8]. Furthermore, long-lived *C. elegans* mutants (Table 1) depend on functional autophagy to maintain their phenotype, since knockout of essential ATG genes, including *atg-18*, abolishes the lifespan-extending effect of mutation in e.g. *daf-2* (Table 1) [8,18].

Thus, WIPI proteins, due to their PI3P-effector function in autophagy, are thought to operate at the interface of autophagy and longevity, linking intracellular clearance to healthy aging. Here, we will discuss the role of human WIPI proteins, as well as their *C. elegans* homologues ATG-18 and EPG-6, in the context of longevity.

**Table 1:** Functional ATG-18 or EPG-6 deficiency in *C. elegans*. Summary of studies either using RNAi or genomic mutations (see column “Functional modification”) in atg-18 (WIPI1/2 in mammals) or epg-6 (WIPI3/4 in mammals), indicated by the column “Gene of interest.” Mutant/transgene *C. elegans* strains are listed in the column “Strain,” and study results are presented in the columns “Phenotype and results” and “Autophagy assessments.” “Ref” stands for references.

Gene of interest	Strain	Functional modification	Phenotype and results	Autophagy assessments	Ref
atg-18	daf-2(e1370) expressing GFP::LGG-1	atg-18 RNAi injection	Abnormal dauer formation in >20% of F1 progeny	Aberrant GFP::LGG-1 localization in seam cells	[16]
atg-18	atg-18(gk378)	Genomic loss-of-function mutation in atg-18	Short-lived phenotype and early onset of age-related locomotory defects		[17]
atg-18	daf-2(e1370); atg-18(gk378)	Genomic loss-of-function mutation in daf-2 and atg-18	Suppression of daf-2 deficiency-dependent long-lived phenotype		[17]
atg-18	atg-18(gk378)	let-363/Tor RNAi feeding	Suppression of let-363/Tor deficiency-dependent long-lived phenotype		[17]
atg-18	atg-18(gk378)	atp-3 RNAi feeding	Suppression of atp-3 deficiency-dependent long-lived phenotype based on mitochondrial respiratory activity		[17]
atg-18	atg-18(gk378)	Genomic loss-of-function mutation in atg-18	Short-lived phenotype	LGG1, PGL, SQST-1, SEPA-1 accumulation in the absence of LGG-1/PGL or LGG-1/SEPA-1 colocalization	[15,31,32]
atg-18	atg-18(gk378)	Genomic loss-of-function mutation in atg-18	Impairment of locomotion, deterioration of muscle fibers		[34]
atg-18	eri-1(mg366)	atg-18 RNAi feeding	Reduced body size (11% of animals), pale appearance, fail to reach adult state within 60 hours		[35]
atg-18	N2 expressing CED-1::GFP	atg-18 RNAi feeding	Accumulation of apoptotic nuclei in the gonad, reduced cell corpse clearance		[37]
atg-18	atg-18(gk378)	Genomic loss-of-function mutation in atg-18	Hyp7 cells (phagocytes) defective to degrade apoptotic Q cell corpses		[38]
atg-18	atg-18(gk378) expressing GFP::ATG-18 and mCherry in Q cells	Expression of GFP::ATG-18 in atg-18 deficient background	GFP::ATG-18 expression rescues atg-18 deficiency-dependent loss of apoptotic Q cell corps clearance, GFP::ATG-18 localizes at the outer surface of engulfed Q cell corpses		[38]
atg-18	atg-18(gk378) expressing GFP::ATG-18(FKKG) and mCherry in Q cells	Expression of PI3P-binding deficient ATG-18 mutant (GFP::ATG-18(FKKG)) in atg-18 deficient background	PI3P-binding deficient GFP::ATG-18 mutant expression does not rescue atg-18 deficiency-dependent loss of apoptotic Q cell corps clearance		[38]
epg-6	epg-6(bp242)	Genomic loss-of-function mutation in epg-6	Reduced lifespan of L1 larvae in the absence of food, slow growth	Accumulation of LGG-1, polyQ, SQST-1, SEPA-1, PGL, LGG-1/PGL colocalization	[15,31,32]
epg-6	eri-1(mg366)	epg-6 RNAi feeding	Reduced body size (23% of animals), pale appearance, fail to reach adult state within 60 hours		[35]

## 2. Regulation of Autophagy

Traditionally, autophagy was considered to occur stochastically, thereby permitting a constant clearance of the cytoplasm resulting in constitutive cellular rejuvenation. Later, it has been recognized that autophagy is also specifically engaged to counteract the accumulation of damaged cellular material, including protein aggregates and dysfunctional mitochondria, thereby continuously fighting the onset of diseases and aging. It is further distinguished between basal and induced autophagy, whereby basal autophagy is considered to occur constitutively at a low level in all eukaryotic cells. Triggered by conserved signaling pathways, basal autophagy levels become elevated upon a variety of cellular insults, such as nutrient and energy shortage, to produce monomers and energy for anabolic processes. Moreover, autophagy is not purely engaged as a self-consumption (greek, auto: self, phagy: eating) mechanism but also critically as a defense mechanism against invading pathogens that escaped the phagosome–lysosome route of destruction [5,6,10,15]. Hence, autophagy is one of the most important mechanisms to secure cellular homeostasis, intrinsic to maintaining the health span of eukaryotic cells.

Since autophagy is a catabolic mechanism activated upon starvation, its regulation is connected to multiple nutrient sensing pathways that converge on initiatory regulators of autophagy that transduce the receiving signal into autophagosome formation and cargo degradation. One of the initiatory regulators of autophagy is the serine/threonine-specific protein kinase ULK (UNC-51-like kinase), harboring multiple site-specific phosphorylation opportunities for upstream kinases. The mTOR (mechanistic target of rapamycin) complex 1 (mTORC1) represents the amino acid sensing unit in mammalian cells. Amino acid availability activates mTORC1, shifting the cellular metabolism towards protein synthesis and cell growth; alternatively, amino acid deprivation inhibits mTORC1, shifting the cellular metabolism towards protein degradation to compensate for starvation. This response is executed because mTORC1, an evolutionarily conserved inhibitor of autophagy [19,20], no longer phosphorylates ULK at certain inhibitory phosphorylation sites.

ULK is further phosphorylated by AMPK (AMP-activated protein kinase), an evolutionarily conserved energy sensor that is activated when low ATP:AMP ratios prevail in the cell. To compensate energy shortage, AMPK phosphorylates ULK at activatory phosphorylation sites to directly initiate autophagy. AMPK further activates autophagy indirectly by inhibiting mTORC1 [21]. Although the signaling network regulation of ULK phosphorylation is far from being understood, it was recognized that ULK activation can lead to the phosphorylation of Beclin 1 in the PI3KC3 complex [19,20], subsequently activating PI3KC3 to produce PI3P [22]. PI3P production is considered to occur majorly at the endoplasmic reticulum, where it is recognized by WIPI proteins that become rapidly recruited to the nascent autophagosome (Figure 2) [14].

## 3. WIPI Members Are Essential PI3P Effectors in Autophagy

The human WIPI (WD-repeat protein interacting with phosphoinositides) members belong to an ancient seven-bladed beta-propeller family expressed among all eukaryotes with representatives from plants to mammals [11,14]. Due to their ancestral property to specifically bind phosphoinositides, the four human WIPI members WIPI1 to WIPI4, along with their relatives, are being referred to as PROPPINs, for beta-propellers that bind phosphoinositides [14,23]. The PROPPIN family is divided into two paralogous groups, one group containing human WIPI1 and WIPI2, and their *C. elegans* homologue ATG-18, and the other group human WIPI3 and WIPI4, and their *C. elegans* homologue EPG-6 [11]. Through homology modeling of human WIPI1 and multiple protein sequence alignment of WIPI homologues it became apparent that the PROPPIN family is defined by the presence of evolutionarily conserved amino acids that cluster at two opposite sides of the beta-propeller [11,14]. One of this cluster of conserved amino acids represents the phosphoinositide binding site and harbors two critical arginines, R227 and R226 in human WIPI1, within an FRRG motif that can be mutated to produce PI3P-binding deficient WIPI mutants [24,25,26,27].

The PI3P-binding property has early been recognized as an essential requirement for WIPI proteins to localize to both phagophores and autophagosomes, first shown for human WIPI1 [11,26,28]. Currently, it is considered that WIPI proteins are rapidly recruited to the site of autophagosome formation upon localized PI3P production at the endoplasmic reticulum (Figure 2). This specific localization has been demonstrated for WIPI1, WIPI2, and WIPI4 [16,28,29], whereas both the PI3P-binding property and the autophagosomal membrane localization of WIPI3 remain uncharacterized [14]. At the phagophore, WIPI members function as PI3P effectors, perhaps in a non-redundant fashion [14], where WIPI2B specifically recruits the ATG12-5/ATG16 complex for subsequent LC3 lipidation (Figure 2) [13]. Both WIPI1 and WIPI2 have been found to further localize to autophagosomes [28], whereas WIPI4 might dissociate from complete autophagosomes (Figure 2) [14]. In this context, WIPI1 was also detected at LAMP1-positive vesicles [28], whereas WIPI2 was not found to colocalize with LAMP2 [29].

Conceptually, the important role of human WIPI members in autophagy is compromised in a variety of age-related human diseases resulting in autophagy dysregulation [12,14]. An aberrant expression of human WIPI genes in many cancer types has early been demonstrated [11], and WIPI mutations have also been identified in many cancer types [14]. However, whether or not WIPI mutations in human cancer represent bystander or driver mutations needs to be defined in future studies. Intriguingly, *de novo* mutations in the WIPI4 gene cause the neurodegenerative disorder SENDA (static encephalopathy of childhood with neurodegeneration in adulthood), a sporadic NBIA (neurodegeneration with brain iron accumulation) subtype [30]. SENDA-causing mutations in the WIPI4 gene result in the synthesis of unstable WIPI4 protein fragments accompanied with a reduced autophagic flux [30]. This suggests that loss of WIPI4 function *in vivo* inhibits autophagic degradation, as previously shown for WIPI1 and WIPI2 *in vitro* [13,29,31]. Importantly, the identification of WIPI4 mutations in SENDA patients provides the first evidence that dysfunctional autophagy causes neurodegeneration in humans [30,32,33,34]. Hence it is of urgent interest to dissect the molecular contribution of WIPI4 in the process of autophagy in normal and pathologically altered neuronal cells.

## 4. The Role of WIPI Homologues in *C. elegans*

The WIPI homologues in *C. elegans*, ATG-18 (homologous to WIPI1 and WIPI2) and EPG-6 (homologous to WIPI3 and WIPI4) [11], are thought to operate at different positions in the autophagy pathway of *C. elegans*, with ATG-18 probably functioning upstream of EPG-6 [16]. This assumption is based on the observation that an *atg-18; epg-6* double mutant *C. elegans* strain is more similar to the phenotype of animals carrying a mutation in *atg-18* alone [16]. The following will summarize available investigations on the function of ATG-18 and EPG-6 in *C. elegans* (Table 1).

Initially, autophagy genes were identified to be essential for correct dauer formation [17], a developmental stage of *C. elegans* characterized by reduced reproductive capacity and entered to outlast unfavorable environmental conditions (e.g. high temperature). Downregulation of *atg-18* using RNAi in adult animals was found to produce more than 20% animals displaying abnormal dauer morphogenesis in the F1 progeny [17]. Animals exhibiting abnormal dauer morphogenesis also showed an aberrant localization of GFP::LGG-1 (LGG1 is the LC3 homologue in *C. elegans*), indicating defects in the progression of autophagy [17]. Even at temperatures that do not promote dauer formation, the progression of autophagy was found to be disturbed in animals carrying a mutation in *atg-18*, indicated by an accumulation of autophagy substrates (Table 1) [16,35].

Strikingly, genomic mutation of *atg-18* was further identified to cause a reduction in the mean and maximum lifespan in *C. elegans* [18,36]. Furthermore, the long-lived phenotype appearing upon functional inactivation of *daf-2* as well as *let-363/TOR* or *atp-3* was suppressed in animals additionally carrying a genomic mutation in *atg-18* (Table1) [18]. Further, it was found that long-lived *daf-2* mutant animals display an upregulation of *atg-18* mRNA levels by 1.5-fold and their motor neurons show increased autophagy, indicating that the lifespan-extending effect of mutation in *daf-2* depends on an upregulation of autophagy [37].

Besides reduction of mean and maximum lifespan, functional ATG-18 deficiency results in a decline of muscle fibers, impaired movement, and premature occurrence of age-related defects in locomotion, resulting in paralysis [18,38]. RNAi-mediated downregulation of *atg-18* was not only performed in the N2 wild-type background, but also in *eri-1*-deficient nematodes [39]. The siRNA-degrading RNase ERI-1 negatively affects RNAi-mediated downregulation of genes [40]. Thus, nematodes carrying a mutation in *eri-1* were found to possess increased sensitivity towards dsRNA [40]. In this context, downregulation of *atg-18* via RNAi was demonstrated to induce growth defects in 11% of all animals [39].

ATG proteins are further thought to be required for apoptotic cell corpse clearance in *C. elegans*, and were found to be reduced upon downregulation of *atg-18* by RNAi as apoptotic nuclei accumulated in the gonad [41]. Moreover, a functional involvement of ATG proteins in the clearance of cell corpses engulfed by phagocytes was studied using live-cell imaging of neuroblasts [42]. During the development of neuroblasts in *C. elegans*, asymmetric cell divisions occur in the Q cell lineage and produce two apoptotic cells (referred to as Q.aa and Q.pp), which are engulfed and then degraded by epithelial hyp7 cells acting as phagocytes [42]. This phagosome-driven degradation requires the function of at least some ATG proteins, since mutation in *atg-18* does not delay the engulfment, but the degradation of apoptotic Q.aa and Q.pp corpses by slowing down phagosome recruitment of RAB-5 and RAB-7, both of which are required for phagosome trafficking and maturation [42]. Furthermore, effective degradation of Q cell corpses was found to depend on the PI3P-binding property of ATG-18, shown by overexpression of a PI3P-binding deficient GFP::ATG-18(FKKG) mutant and its inability to rescue the *atg-18* mutant phenotype (Table 1) [42]. In contrast, introducing PI3P-binding competent GFP::ATG-18 into the *atg-18* mutant background rescued efficient Q cell corpse degradation [42]. Thus, ATG-18 functions in the degradation of engulfed apoptotic cells, although it remains unclear, whether or not this represents an autophagy or non-autophagy function of ATG-18 in *C. elegans* [43].

Finally, the consequences of functional epg-6 inactivation were also investigated through genomic *epg-6* mutations introducing stop codon encoding sequences [16,35]. These epg-6 mutant strains grew slowly and displayed a reduced mean and maximum lifespan of L1 larvae under starvation conditions [16,35]. These features were attributed to deficient autophagy, since mutation in *epg-6* caused an accumulation of LGG-1 [36] and early autophagic structures, as well as autophagy substrates [16]. In addition, it was demonstrated that downregulation of *epg-6* in an *eri-1*-deficient background induces growth defects in 23% of all animals (Table 1) [40].

**Figure 3:**
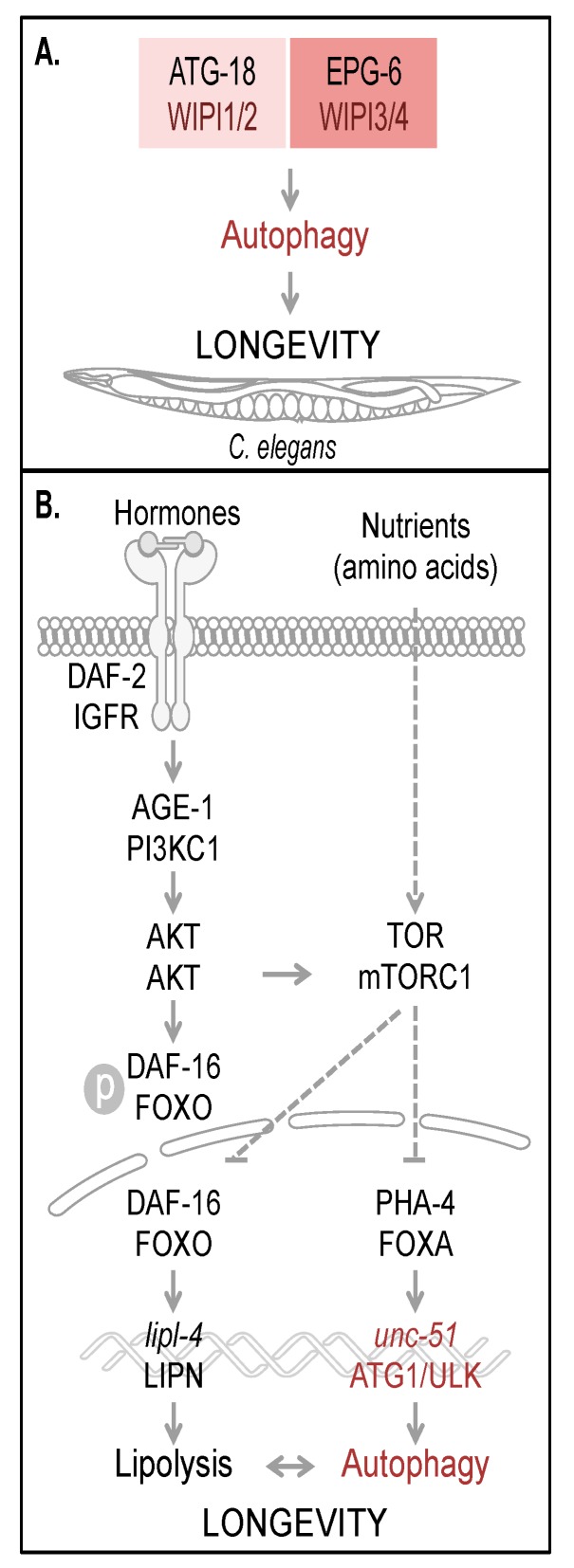
Autophagy and longevity control in *C. elegans*. The four human WIPI proteins are represented by two homologues, ATG-18 (homologue of WIPI1 and WIPI2) and EPG-6 (homologue of WIPI3 and WIPI4) in *C. elegans*, both of which are considered to be required for autophagy-controlled longevity (**A**). Hormones and nutrients (e.g. amino acids) control both autophagy and longevity in *C. elegans* (**B**). Upon hormone binding to and dimerization of DAF-2, the *C. elegans* orthologue of mammalian insulin/IGF-1 receptor (IGFR), AKT (also AKT in mammals) is activated via AGE-1 (PI3KC1 in mammals) to phosphorylate the transcription factor DAF-16. Phospho-DAF-16 is unable to localize to the nucleus. In the absence of AKT-mediated phosphorylation, DAF-16 localizes to the nucleus and fulfills its transcriptional transactivation activity on a large subset of genes controlling stress resistance and survival, by upregulation of mediators required for detoxification, anti-inflammation and lipolysis (lipl-4, LIPN in mammals). DAF-16 is further critically controlled by TOR (mTOR in mammals, a well-known target of AKT-mediated signaling). Upon nutrient availability (e.g. amino acids), TOR inhibits both transcription factors DAF-16 and PHA-4 (FOXA in mammals). Subsequently unc-51 (ATG1 in yeast and ULK in mammals) transcription is inhibited, preventing full autophagic activity. Interdependently, lipolysis and autophagy control longevity in *C. elegans.* The schematic drawings of *C. elegans*, plasma membrane, receptor, nuclear envelope and DNA were obtained from Motifolio.

From this it was deduced that both ATG-18 as well as EPG-6 have an essential function in autophagy and the degradation of engulfed apoptotic cell corpses in *C. elegans*. Hence the functional contribution of both ATG-18 and EPG-6 in autophagy is considered to define the lifespan of *C. elegans* and to contribute to longevity (Figure 3A).

## 5. Regulation of Autophagy and Longevity in *C. elegans*

The regulation of autophagy tightly interconnects multiple nutrient sensing pathways, which ensures that in both times of food deprivation and times of sufficient nutrient supply, cellular homeostasis can be maintained to facilitate survival and to promote longevity [44]. In response to starvation conditions (referred to as dietary or caloric restriction), certain key regulatory factors are activated to upregulate the transcription of essential target genes driving catabolic processes such as lipolysis and autophagy (Figure 3B) [45,46]. Interestingly, mutations causing a metabolic shift towards catabolic pathways, mimicking calorie restriction, were found to promote longevity by an upregulation of autophagy [17,18]. In this context, two signaling pathways that regulate lifespan in *C. elegans*, the conserved insulin/IGF-1 (insulin-like growth factor 1) and TOR pathway, are considered to act through autophagy regulation on longevity (Figure 3B) [47].

Two of the first identified long-lived *C. elegans* mutants carry mutations in genes involved in the insulin/IGF-1 signaling pathway: *daf-2* and *age-1* (Figure 3B) [48,49]. Mutation in *daf-2*, which is the *C. elegans* homologue of the mammalian insulin/IGF-1 receptor (IGFR), was demonstrated to more than double the lifespan, which requires the activity of a second gene, *daf-16* [48]. Upon hormone binding to DAF-2, subsequent signal transduction results in the inhibition of the FOXO transcription factor DAF-16 [47]. DAF-2, when it is activated, phosphorylates and thereby activates AGE-1, the *C. elegans* homologue of mammalian PI3KC1, followed by activation of PDK-1 and AKT-1/2 [47]. Activation of this pathway leads to the phosphorylation of DAF-16 (Figure 3B), which, when phosphorylated, is unable to translocate into the nucleus, where it functions as a site-specific transcription factor, globally regulating lifespan [47]. Thus, mutations in either DAF-2 or AGE-1 cause a constitutive activation of DAF-16 and transcriptional upregulation of its target genes, involved in stress response and survival mechanisms, promoting anti-inflammation, lipolysis, and detoxification [47]. Beyond that, DAF-16 activity is also regulated by additional mechanisms sensing nutrient conditions through the TOR signaling pathway, inhibiting DAF-16 in the presence of nutrients and liberating DAF-16 inhibition during starvation (see below).

Autophagy was early demonstrated to be required for lifespan extension achieved by mutating *daf-2*, since downregulation of *bec-1* via RNAi reverted the long-lived phenotype of *daf-2* mutant nematodes to N2 wild-type animals [17]. However, the absence of DAF-16 alone is not considered to boost autophagy for promoting longevity [45], but an increase of atg-18 mRNA abundance has been recognized in the daf-2 deficient background [37].

TOR is a key regulatory factor in *C. elegans*, activated in response to starvation conditions with impact on many intracellular metabolic processes, including protein synthesis, hypoxia adaptation, and several other processes linked to metabolism and growth [46]. According to TOR regulation in various organisms, functional inactivation of LET-363, the *C. elegans* homologue of mTOR, is sufficient to promote longevity in *C. elegans* by upregulating the activity of UNC-51, the *C. elegans* homologue of ULK, through PHA-4/FOXA-dependent *unc-51* expression (Figure 3B) [47]. Importantly, lifespan extension achieved by TOR inactivation or dietary restriction was demonstrated to depend on autophagy, since both PHA-4/FOXA inhibition and ATG gene inactivation reverted to the long-lived phenotype under these conditions [45,47].

Two TOR complexes have been identified in *C. elegans*, TORC1 and TORC2; however, it is unclear in which of these complexes TOR regulates PHA-4/FOXA-dependent *unc-51* gene expression [47]. Nevertheless, TORC1 was demonstrated to inhibit DAF-16/FOXO, thereby communicating with the insulin/IGF-1 pathway (Figure 3B) [47]. Of note, the function of PHA-4/FOXA as a transcription factor was found to be highly context-dependent, since it fulfills essential functions in the development of the pharynx during embryogenesis, while in adult nematodes it provides lifespan extension by inducing autophagy [46].

Since reproductive capacity and longevity can be considered as two contrary energy-consuming concepts, both should require common regulatory factors. DAF-16/FOXO and PHA-4/FOXA represent two of the main regulatory components with impact on transcriptional programs that integrate signals from germline cells influencing metabolic processes [50,51]. In this context, autophagy, in its regulation and progression, is thought to be strongly interconnected with lipid metabolism and reproductive growth [52]. In germline-less nematodes, TOR is one of the key regulators of both lipid metabolism and autophagy. Here, the activity of the lipase *lipl-4*, permitting lipolysis, and autophagy seem to be interdependent (Figure 3B) [53]. Germline removal was shown to upregulate the expression of *lipl-4*, a process that depends on DAF-16/FOXO, and overexpression of LIPL-4 alone is sufficient to promote longevity in germline-less and wild-type nematodes [51,52]. However, lifespan extension achieved by promoting lipid metabolism also requires the activity of ATG genes and PHA-4/FOXA, suggesting TOR as a common regulator of both autophagy and lipid metabolism in *C. elegans* [52]. Based on this, lipid homeostasis contributes to the lifespan-extending effect of upregulated autophagy observed in germline-less nematodes [53].

## 6. Conclusions

WIPI proteins can be considered as essential PI3P effectors in the process of autophagy-driven longevity. This assumption is based on early findings demonstrating that (i) human WIPI proteins fulfill important functions in decoding the essential PI3P signal to initiate autophagy, (ii) human WIPI protein dysregulation, e.g. WIPI4 mutation in SENDA, is causative for neurodegeneration, and (iii) WIPI homologues in *C. elegans* are required for autophagy and lifespan extension. From this it is tempting to speculate that WIPI proteins might represent putative targets for anti-aging therapies that focus on the modulation of autophagy.
